# The impact of digital transformation on corporate total factor productivity

**DOI:** 10.3389/fpsyg.2022.1071986

**Published:** 2022-12-07

**Authors:** Na Li, Xiaohong Wang, Zeren Wang, Xiangyu Luan

**Affiliations:** ^1^School of Management, Harbin Institute of Technology, Harbin, China; ^2^School of Business, China University of Political Science and Law, Beijing, China

**Keywords:** digital transformation, total factor productivity, technical cooperation, ESG performance, corporate productivity

## Abstract

**Introduction:**

Corporates need to break through the dilemma of system and efficiency with the help of digital transformation in the digital economy era. This paper aims to examine the influence of digital transformation on corporate total factor productivity by investigating whether and how corporate technical cooperation and ESG performance mediate and moderate the relationship between them.

**Methods:**

This study choose Chinese A-share listed manufacturing firms from 2016–2020 as the research sample and use the FGLS regression model to test the proposed hypotheses.

**Results:**

Results show that digital transformation has a positive effect on corporate total factor productivity, and this positive impact is more pronounced when corporates have higher ESG performance. Corporate technical cooperation plays a mediating role between digital transformation and total factor productivity. ESG performance also plays a positive moderating role in the relationship between digital transformation and corporate technical cooperation.

**Discussion:**

Our results contribute to the literature on digital transformation and corporate total factor productivity at the micro-corporate level. Further, our findings offer insights to decision-makers and regulatory bodies regarding the current practices of digital transformation and its potential economic impact.

## Introduction

China’s manufacturing industry has made great strides in development in recent years. However, with the loss of the demographic dividend and the impact of the new global epidemic, how to form an overall competitive advantage for manufacturing enterprises has become an important proposition for societies and academics in various countries to consider. Modern economic growth theory shows that total factor productivity (TFP) is the power source for corporate management and development. As the marginal productivity of traditional factors such as resources and labor decreases, the growth of corporate TFP stagnates, and economic growth slows down. How to realize the sustainable growth of corporate by improving TFP is very important (Li et al., 2022).

In theoretical research, several scholars pay attention to the impact of technological progress on productivity. Viollaz (2019) pointed out that ICT application improves corporate productivity. Pieri et al. (2018) proposed the joint impact of R&D investment and ICT on productivity. The above research provides a rich basis for explaining the impact of information technology on TFP. At the same time, many countries have adopted digital economy development strategies such as “Industrial Internet” and “Industrialization 4.0.” The aim is to promote the manufacturing industry’s digital transformation (DT) to achieve economic transformation DT relies on new technologies to improve the corporate technological level and management efficiency from multiple aspects of technology, information, and platforms to remove barriers to corporate TFP. As a result, DT is gradually becoming a vital breakthrough point for innovative change in global enterprises and a critical theoretical frontier issue that needs urgent attention. Many scholars have studied the impact of DT on corporate economic performance (Khin and Ho, 2019) or corporate value (Rahmati et al., 2021). Few scholars have found that DT can improve corporate TFP by improving management efficiency and technical level, and this promoting effect only exists in small and medium-sized high-tech enterprises (Zeng and Lei, 2021). The existing studies found that despite theoretical support, there is still a significant amount of unexplored research on DT and TFP in micro-economies. Chinese corporate DT is still in the early stage of development, and its impact on TFP, especially its mechanism, has not been studied in depth. Therefore, it is necessary to explore the mechanisms of DT and corporate TFP in China.

Meanwhile, under the background of a new round of scientific and technological revolution and industrial reform, the way to improve the corporate TFP is bound to change under DT. Existing studies have confirmed that the core competitiveness of the manufacturing industry currently lies in innovation plus complementary assets (Teece, 1986). Therefore, with the technological complexity increase, corporate innovation activities are no longer a simple technological innovation behavior that depends on internal resources but more on multiple innovation subjects interaction. Firms urgently need to enhance their innovation ability through technical cooperation (TC) to improve TFP (Wang et al., 2018). Significantly, DT can help corporate break through organizational boundaries and absorb external heterogeneous resources in a deeper and broader range. As a result, corporates have realized the sharing of innovation resources, accelerated the speed of corporate R&D through TC, and improved the corporate TFP. It can be seen that TC is an important path for corporate to improve TFP under the DT. Existing studies have gradually focused on information technology in corporate TC (Wang et al., 2018). However, there is still a lack of empirical analysis on the impact of DT on enterprise TFP from the perspective of TC. Therefore, this paper will explore the mechanisms of the impact of corporate DT on TFP from a TC perspective to bridge the little literature gap.

Likewise, under the concept of sustainable development, corporates are paying more attention to the values of their stakeholders. Creating integrated economic, social, and environmental value based on sustainable development has become the direction corporate’s pursue. ESG is a new corporate sustainability concept on how environmental, social and corporate governance can be harmonized and is an essential vehicle for corporate non-financial information disclosure. High ESG performance makes it easier for corporates to gain the trust of external technical partners and prevent the management’s short-sighted behavior from forming a catalyst in the process of DT to promote TC and TFP. However, regardless of the mechanisms through which ESG exerts its influence on corporate. It cannot ignore its moderating effect in the process of DT influence on TC and TFP. Most existing ESG studies focus on the impact on financing constraints (Zhang et al., 2022), innovation levels (Xu et al., 2020), or economic performance (Yu et al., 2021). Empirical studies are also needed to support the moderating role of ESG in the corporate business process.

In summary, this paper aims to examine the influence of DT on TFP by investigating whether and how corporate TC and ESG performance (ESG) mediate and moderate the relationship between them. The study’s contribution to the literature is threefold. First, existing research is less focused on the impact of DT on TFP in micro-enterprises. Our study extends the current understanding of the antecedents of corporate TFP by showing that DT is an essential driver of corporate TFP. Although many scholars have explored the impact of DT on corporate innovation or performance (Nambisan et al., 2019; Zomer et al., 2020), it has largely ignored the corporate TFP. Therefore, our study reveals the key role of digital elements in the corporate productivity discussion. Second, we found that corporate TC plays a mediating role between DT and corporate TFP. Although previous research has investigated the influence of digital technologies on corporate collaborative innovation (Wang et al., 2018). To the best of our knowledge, this study is one of the first attempts to examine the impact mechanism of DT on corporate TFP achieved through the mediating effect of corporate TC. Third, our study offers a novel theoretical contribution by investigating how ESG interacts with DT in corporate TC and TFP. We found that the positive impact of DT on TC and TFP is more pronounced when firms have a high level of ESG. In doing so, this study’s findings support the social responsibility theory in the context of the current digital ecosystem.

The remainder of the paper is structured as follows. Section 2 discusses a literature review and hypothesis development. Section 3 describes the data, sample, and methods. Section 4 presents the empirical results. Section 5 discusses the results and concludes the study.

## Literature review and hypothesis development

Existing research suggests that DT can promote TFP by facilitating technological progress and improving managerial efficiency. Pan et al. (2022) found a non-linear effect between the digital economy and regional TFP. Brynjolfsson and Hitt (2002) found that firms are more productive when investment in ICT capital is combined with job restructuring and the use of skilled labor in a study using data from 527 large U.S. firms over 1987–1994. Zeng and Lei (2021) argued that DT enhances TFP by improving business management efficiency, but this contribution is only found in small and medium-sized high-tech corporates. Similar studies have examined the impact of ICT on corporate productivity. Mittal and Nault (2009) studied the impact of ICT on output. Viollaz (2019) found that ICT can increase corporate productivity. Pieri et al. (2018) focus on the collective impact of R&D investment and ICT on productivity. Mohnen et al. (2018) find that ICT is complementary to R&D and organizational innovation as joint investment leads to higher TFP in the Netherlands. Ballestar et al. (2020) extended this literature to include complementarities linked to using robots and e-commerce using Spanish firm-level data in a recent study. They found positive complementarities between the combination of robotics and a composite measure of e-commerce. DT relies on new technologies such as blockchain and big data to improve the corporate technological level and management efficiency from multiple aspects of technology, information, and platforms to remove barriers to corporate TFP. The existing studies found that despite theoretical support, there is still a significant amount of unexplored research on DT and TFP at a firm level.

### DT and TFP

Information is the processed higher-order form of data. The keys of DT are information collection, data processing, and the application of digital technology to aid decision-making (Wu et al., 2019). IPT theory suggests that corporates must be able to collect, decompose, synthesize, and disseminate high-quality information to cope with operational uncertainty and improve their decision-making capabilities (Galbraith, 1973). The theory emphasizes the results of organizational information processing and the increased ability to achieve organizational goals. In this study, DT enhances the corporates’ ability to process information. Moreover, information processing is the practical means DT empowers corporate TFP. Therefore, this paper focuses on the mechanism of the impact of DT on TFP based on IPT theory.

First, DT can improve TFP by upgrading the traditional industrial sector. The embedded industrial internet application connects business processes, management systems, and supply chain data. Corporates can achieve precise services and assist management decisions through data penetration and intelligent analysis of the entire chain. It enables refined management of the whole product life cycle and effectively addresses management issues and business operations. In addition, DT allows corporates to analyze and manage strategy through the lens of data. It will enable corporates to act more proactively and make more proactive decisions. And corporates can accurately predict consumer tendencies and business risks based on data such as product transactions and user reviews deposited on digital platforms (He et al., 2019). In particular, Commander et al. (2011) argued that the complementarity between computer investment and other allied investment forms, such as organizational change, has been emphasized. The embedded industrial internet application connects business processes, management systems, and supply chain data. Firms can achieve precise services and assist management decisions through data penetration and intelligent analysis of the entire chain. Such organizational complements, leading to improvements in better-quality goods and improved service speed, are important in explaining why particular firms have reaped productivity benefits and others have not.

Second, traditional trading methods are limited by geographical space. So there is a natural market segmentation problem between supply and demand. Firms can use e-commerce platforms and mobile payment tools to make otherwise impossible transactions and Nguyen et al. (2018) argued that DT enhances corporate production decisions with the more efficient collection and production-and of operation-related information. The precise matching of supply and demand through big data technology solves the coordination problem and improves corporate resource allocation efficiency. Thereby conducive to expanding the corporate product sales scale, realizing scale economies and promoting TFP.

Third, DT serves corporate production decisions with the more efficient collection and information processing related to production and operations (Nguyen et al., 2018). Muninger et al. (2019) found that digital technology can facilitate rapid decision-making and knowledge flows across teams within the corporate from an organizational capabilities perspective through a survey of managers in Western Europe and the United States. Corporates can also collaborate with suppliers based on data analysis to achieve personalized design, flexible production, intelligent warehousing, and just-in-time delivery resulting in a zero-inventory production model (Zeng and Lei, 2021). DT promotes the interconnection of data between systems and platforms by upgrading the manufacturing of traditional manufacturing plants to intelligent workshops, thus realizing real-time analysis and scientific decision-making in production. Additionally, DT can alleviate problems such as backward industrial design tool software and low R&D efficiency. For example, corporates can simulate various physical parameters accurately through simulation design tools. It can improve the accuracy of R&D and realize data-driven intelligent production. Moreover, Commander et al. (2011) also empirically found a strong positive association between investments in ICT capital and productivity using manufacturing firm-level data in Brazil and India. Based on this, we propose the following:

*H1*: DT has a significantly positive effect on TFP.

### DT and TC

TC refers to the systematic collaboration between corporates and other organizations for “technological development” and “technological problem solving” about new products or processes (Subramanian et al., 2013). However, geographical distance is a big obstacle in TC. Black and Lynch (2001) found evidence of positive complementarities between ICT and workplace innovations based on the sample of more than 3,000 private establishments with more than twenty employees by the EQW National Employers Survey in America. It can be seen that DT effectively breaks down economic activity spatial constraints, reduces information communication and replication costs, and reshapes the way value is co-created between corporates and other organizations. It enhances knowledge diffusion and spillovers, increasing the innovation factors among collaborating actors’ distribution efficiency and the immediacy of TC (Dubey et al., 2019). Polder et al. (2010) provide further evidence that broadband internet access can acquire new knowledge inputs for sharing knowledge between corporate and other partners through a Dutch case study. Moreover, several cases proved that social media can be used to support knowledge sharing and open innovation (Brandtzaeg et al., 2016). The multiplier effect of online platforms such as the Internet or social media can effectively integrate different organizations in a short time. It expands the knowledge reserve and promotes knowledge sharing among cooperation subjects in TC.

DT has brought new models and opportunities to corporate. However, these innovation opportunities require new innovation capabilities. Firms’ pre-existing technological reserves may not match them, forcing corporate to constantly seek external help for TC to replenish and update their technical reserves. In addition, DT has led to disruptive business models (Broekhuizen et al., 2021). It has led to an increasingly complex innovation ecosystem, pushing the breadth and depth of collaboration between corporates and other organizations to a higher level. The uncertainty of the external environment for corporate innovation activities is increasing, making it more expensive to gather information and more difficult to predict future trends. In this background, corporates need to deepen TC to gain a competitive advantage. For example, corporates can promote the decentralization of R&D activities through global collaborations (Szalavetz, 2019). Further, build integrated innovation network platforms and improve the judgment of technological trends to resist external competitive pressures. Based on this, we propose the following:

*H2*: DT has a significantly positive effect on TC.

### Mediating effect of TC

On the one hand, as analyzed above, DT effectively breaks down the barriers of space limitations of TC and strengthens the connection between corporates and different organizations. Instant messaging, video conferencing, and cloud platforms enable knowledge, data, and ideas to be shared more freely and corporate more collaboratively organized, thus facilitating TC between corporates and different organizations. On the other hand, a key driver of TFP is technological progress driven by innovation. Using the data from the micro and small corporates in South Africa, Gaglio et al. (2022) found that selected digital communication technologies have a significant effect on innovation and that innovation conditional based on the technologies has a positive effect on corporate productivity. Crespi and Zuniga (2012) found evidence that innovation has a significant effect on productivity through a sample of six Latin American countries. TC is one of the important forms of corporate innovation. Antonioli et al. (2017) propose that collaboration innovation can overcome barriers in knowledge and finance to share the risks and innovation costs. TC facilitates the dissemination and sharing of knowledge among different innovation actors (Fabrizio, 2009). It can combine and integrate more inter-organizational resources to help corporates overcome technological challenges. Firms can reduce production costs and improve product quality through joint research with corporates in the same industry and academic or research institutions (Singh et al., 2022). As a result, corporates can enjoy increased profits from expanded production, further deepening the impact of innovation on TFP. In addition, TC helps other organizations’ innovations diffuse into their corporate through these networks. It enables corporate to learn to generate absorptive capacity to identify, integrate, and implement knowledge, thus promoting TFP through filtering out ideas with insufficient technological content and developing advanced technologies for other corporates to imitate. Thus, they enhance their ability to innovate and increase TFP. Therefore, TC plays a crucial mediating role in DT promoting TFP. Based on this, we propose the following:

*H3*: TC plays a mediating role between DT and TFP.

### Moderating effect of ESG

ESG performance is an acronym for Environment, Social and Governance. It represents the degree of corporate involvement in CSR activities. Although DT is a vital driver for corporate transformation and upgrading, there is still a more significant risk. Based on the principal-agent theory, managers are often reluctant to invest in DT. However, ESG effectively mitigates principal-agent conflicts through incentive constraints and ensures the separation of corporate decisions management and control (Chouaibi et al., 2021). At the same time, investors are more willing to shift their capital to corporate with higher sustainability ratings, forcing managers to turn from self-interested behavior to sustainable strategies. It helps corporate consolidate their existing digital resource base and improve their TFP through DT. Social responsibility theory suggests that enterprises and stakeholders are a “community of destiny.” It means that corporate value is realized by meeting stakeholder expectations. ESG enables corporate to establish more extensive and solid relationships with multi-stakeholder subjects (Chouaibi et al., 2021). Carmer (2010) argued that corporate social responsibility could acquire diversified knowledge resources by building external knowledge networks, thus helping them explore new market opportunities and promote subsequent TC in the process of DT. ESG conveys the concept of corporate sustainability to the outside world and establishes a good image of social responsibility for the corporates (Chouaibi et al., 2021). It helps corporate win the trust of their technology partners and attracts other organizations to collaborate with them in technology (Fatemi et al., 2015). Further enhances the facilitation effect of DT on TC.

Branco and Rodrigues (2006) argued that ESG is conducive to enhancing the firm’s reputation and strengthening its stakeholders’ interactions. It increases internal and external social capital and promotes the gathering of all types of resources, such as technology, information, capital, policy, and professional talent. From this dimension, a high level of ESG is likely to contribute to the maximum effectiveness of DT. Moreover, using the MSCI ESG rating data and financial variables, Giese et al. (2019) argued that ESG was transmitted to corporate valuation and performance through their systematic risk profile (lower costs of capital and higher valuations) and their idiosyncratic risk profile (higher profitability and lower exposure to tail risk). Lins et al. (2017) pointed out that corporate with good ESG performance has relatively stable market capitalization, share prices are more resilient in the face of extreme risks, and have a lower financial burden in the United States. Broadstock et al. (2021a) found that ESG can improve managed portfolio performance and reduce risk. The above studies prove that higher ESG will create a good condition for DT to improve TC and TFP. In particular, Cahan et al. (2015) find that excellent ESG performance generates favorable publicity. It can play an “insurance” role when corporates are hit by negative events, thus reducing the losses and alleviating the financing constraints in the process of DT. Therefore, we argue that ESG will enhance the positive relationship between DT and TC as well as DT and TFP. Based on this, we propose the following:

*H4*: ESG will enhance the positive relationship between DT and TC.

*H5*: ESG will enhance the positive relationship between DT and TFP.

**Figure 1:**
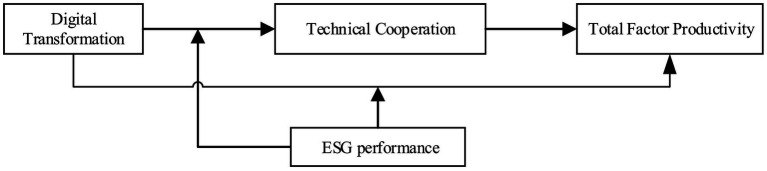
Theoretical framework.

Overall, corporates are more likely to leverage DT to establish TC to achieve higher economic TFP. Considering the mediating role of corporate TC, this study highlights that the indirect effects of DT on corporate TFP through TC will be enhanced as corporate ESG grows. Moreover, the positive impact of DT on TFP is more pronounced when firms have higher proportions of ESG. And the following hypotheses are proposed in this study. Furthermore, a moderated mediation effect exists when a mediation process is dependent on a moderating variable (Yuan et al., 2020). Based on the above structural logic, we constructed the theoretical framework (see Figure 1).

## Materials and methods

### Sample and data collection

The source of the empirical analysis is listed as A-share manufacturing corporates in China. DT data are obtained by collecting and organizing annual reports for textual analysis. Other corporate-level data are from the Wind, CNRDS, and CSMAR databases. After excluding corporates with special treatment and missing data, the final valid data consisted of 4,222 firm-year observations.

### Measures

#### Dependent variable

TFP is the dependent variable. Several methods for estimating micro-firm TFP have been analyzed in the literature. The more widely used methods are OLS, Olley-Pakes, Levinsohn-Petrin (LP) and others. Consider that the LP method can more sharply capture the micro-firms response to productivity changes. It avoids data truncation and endogeneity problems. Therefore, we adopted the LP method and tested the accuracy of the results in a later robustness check by OLS.

#### Independent variable

**Figure 2:**
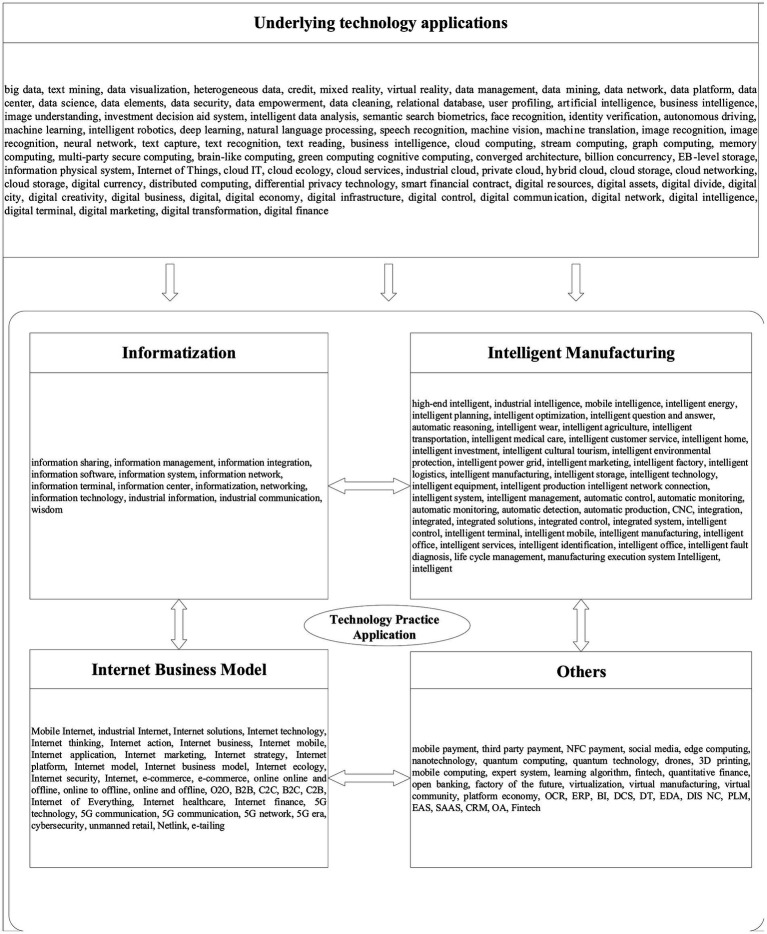
DT feature word.

The independent variable in this paper is DT. Following previous studies (Gong and Ribiere, 2021; Meng et al., 2022), we construct the DT index by conducting a dictionary-based text analysis of the listed firms’ annual reports. First, we collect the annual reports of listed manufacturing corporates from 2016 to 2020. Second, we determine the DT feature words from “underlying technology application” (UTA) and “technology practice application” (TPA). UTA mainly focuses on the embedding of digital technology. TPA applies specific business scenarios in information technology, intelligent manufacturing, and Internet business models as shown in Figure 2. Third, we use the weight of DT feature words in the annual reports of each manufacturing corporate in the sample to the total number of keywords appearing for all manufacturing corporates in that year as a metric (Li et al., 2022). The formula is


(1) $$q1=a_{it}/\overset{i}{\underset{t}{\sum }}ait$$

Where *q*1 is DT, *i* is corporate code, *t* is the year, and a is the number of keywords for each corporate.

#### Mediating variable

The mediating variable in this study is TC. TC is a direct collaboration between corporate and other organizations around a technological innovation project. It typically takes the form of joint technological inventions, and the direct output is a joint patent application (Subramanian et al., 2013) In addition, considering that the number of patents granted numbers can ensure the inventiveness and novelty of the patents, so we choose the enterprise joint patent grant number measured TC.

#### Moderating variable

The mediating variable is ESG. This paper focuses on ESG performance scores as disclosed by Bloomberg. This score can be broken into environmental, social responsibility, and corporate governance. The higher the score, the higher the degree to which the corporate is fulfilling its respective responsibilities.

#### Control variables

Several variables that may influence TFP are included as control variables: corporate size (Size). The corporate age (Time). The capital structure (Lev). The first major shareholder shareholding ratio (FMS). Corporate R&D (RD). The net interest rate on total assets (ROA). Inventory Turnover (Inv). Year fixed effects (Year) and region fixed effects (Region). Each variable is defined as shown in Table 1.

**Table 1:** The results of descriptive statistics.

Variable type	Variable symbol	Definition
Dependent variable	TFP	The corporate TFP was estimated by the Levinsohn-Petri (LP) method
Independent variable	DT	DT Index
Mediating variable	TC	Logarithm of the number of joint patents granted
Moderating variable	ESG	ESG scores from Bloomberg
Control variables	Time	Time of establishment of each corporate
	FMS	Percentage of shareholding of the largest shareholder
	Size	The logarithm of fixed assets
	RD	R&D expenses/operating revenue
	Inv	Inventory turnover rate
	ROA	Net profit/mean total assets
	Lev	Total liabilities / total assets
	Year	Year fixed effects
	Region	Region fixed effects.

Table 2 shows the descriptive statistics of the variables. The average value of DT is 0.102, the minimum value is 0, and the maximum value is 1.703, indicating that corporate DT is generally low. We use Pearson’s correlation coefficient matrix to analyze the correlations between variables. We also perform a multicollinearity test on the variables; the variance inflation factors are less than 10. Therefore, we can conclude that the variables selected in this study are not multicollinearity.

**Table 2:** The results of correlation analysis and multicollinearity test.

Variable	1	2	3	4	5	6	7	8	9	10	11
1. TFP	1										
2. TC	0.263***	1									
3. DT	0.037**	0.109***	1								
4. ESG	0.585***	0.227***	0.032**	1							
5. FMS	0.163***	0.0150	−0.023	0.143***	1						
6. ROA	0.132***	0.0190	−0.017	0.133***	0.151***	1					
7. Inv	0.360***	0.041***	−0.003	0.113***	0.057***	0.063***	1				
8. RD	−0.354***	0.052***	0.244***	−0.108***	−0.094***	−0.120***	−0.248***	1			
9. Lev	0.517***	0.141***	0.046***	0.218***	0.032**	−0.310***	0.158***	−0.206***	1		
10. Size	0.792***	0.229***	−0.085***	0.544***	0.115***	0.004	0.225***	−0.303***	0.470***	1	
11. Time	0.185***	0.069***	−0.084***	0.141***	−0.007	0.000	0.007	−0.118***	0.083***	0.207***	1
Mean	11.007	0.526	0.102	8.729	31.628	4.379	3.959	4.551	38.166	20.445	19.746
Std	1.101	1.035	0.126	11.906	13.145	7.182	3.462	3.789	17.398	1.308	5.324
Min	8.086	0.000	0.000	0.000	2.870	−71.268	0.018	0.000	1.427	15.515	5.000
Max	14.378	6.805	1.703	60.744	83.410	47.752	62.468	57.308	99.007	25.258	62.000
VIF		1.10	1.11	1.51	1.05	1.23	1.11	1.28	1.53	1.95	1.06

*, ** and *** represent the significance levels of 10, 5 and 1%, respectively. Same below.

### Empirical model setting

The following model is set to test the path and boundary conditions of the effect of DT on TFP. Eq. (2) tests the effect of DT on TFP, Eq. (3) tests the effect of DT on TC, Eq. (4) tests the mediating role of TC, and Eq. (5) tests the moderating role of ESG:


(2) $$\mathrm{TFP}=\beta 0+\beta 1DT+\overset{m}{\underset{1}{\sum }}\beta mCi,t+\varepsilon i,t$$

(3) $$\mathrm{TC}=\beta 0+\beta 1DT+\overset{m}{\underset{1}{\sum }}\beta mCi,t+\varepsilon i,t$$

(4) $$\mathrm{TFP}=\beta 0+\beta 1DT+\beta 2TC+\overset{m}{\underset{1}{\sum }}\beta mCi,t+\varepsilon i,t$$

(5) $$\begin{array}{l} \mathrm{TFP}/\mathrm{TC}=\beta 0+\beta 1DT+\beta 2ESG+\beta 3DT\times \\ \;\;\;\;\;\;\;\;\;\;\;\;\;\;\;\;\;\;\;\;\;\;\;\;\;\;\;\;ESG+\overset{m}{\underset{1}{\sum }}\beta mCi,t+\varepsilon i,t \end{array}$$

## Empirical results

### Regression results

Heteroscedasticity tests are performed on the model residuals before regression analysis. The results show that each model rejects the original hypothesis of the modified Wald test at the 1% level, indicating that the model has a heteroscedasticity problem. So we use the feasible generalized least-squares method for estimation to eliminate this problem. The regression results are shown in Table 3.

**Table 3:** Regression results.

	TFP	TC	TFP	TC	TFP
	(1)	(2)	(3)	(4)	(5)
DT	0.882***	0.558***	0.852***	0.244***	0.620***
	(23.97)	(8.36)	(22.89)	(3.22)	(14.53)
TC			0.067***		
			(17.13)		
ESG				0.005***	0.018***
				(5.27)	(35.95)
DT × ESG				0.037***	0.015***
				(4.37)	(4.73)
FMS	0.003***	0.001	0.003***	0.001	0.003***
	(10.77)	(1.19)	(9.78)	(0.65)	(9.96)
ROA	0.026***	0.003***	0.026***	0.002***	0.022***
	(38.91)	(5.98)	(38.02)	(3.29)	(34.33)
Inv	0.047***	−0.002	0.048***	−0.001	0.050***
	(30.45)	(−1.36)	(31.14)	(−0.13)	(36.87)
RD	−0.027***	0.014***	−0.029***	0.013***	−0.032***
	(−20.88)	(7.75)	(−21.54)	(7.13)	(−25.73)
Lev	0.014***	0.002***	0.014***	0.002***	0.014***
	(47.20)	(4.85)	(45.72)	(4.82)	(51.84)
Size	0.527***	0.100***	0.516***	0.066***	0.425***
	(126.49)	(17.31)	(124.74)	(10.59)	(98.35)
Time	0.005***	0.004***	0.004***	0.002*	0.004***
	(7.28)	(3.14)	(6.25)	(1.86)	(6.04)
Year	YES	YES	YES	YES	YES
Region	YES	YES	YES	YES	YES
_cons	−0.641***	−2.061***	−0.412***	−1.373***	1.250***
	(−7.73)	(−17.33)	(−4.93)	(−11.02)	(14.73)
N	4,222	4,222	4,222	4,222	4,222
Wald chi2	53439.560***	825.840***	55028.070***	842.860***	76253.230***

In the regression (1) results, DT is significantly positively related to TFP (*β* = 0.882, *p* < 0.01). In the regression (2) results, DT is significantly positively related to TC (*β* = 0.558, p < 0.01). In the regression (3) results, TC is significantly positively related to TFP (*β* = 0.067, p < 0.01), and DT is significantly positively related to TFP (*β* = 0.852, *p* < 0.01), indicating that TC partially mediates the relationship between DT and TFP. In the regression (4) results, DT × ESG is significantly positively related to TC (*β* = 0.037, *p* < 0.01). It shows that ESG can play a positive moderating role between DT and TC. In the regression (5) results, DT × ESG is significantly positively related to TFP (*β* = 0.015, *p* < 0.01). It shows that ESG can play a positive moderating role between DT and TFP. *H*1, *H*2, *H*3, *H*4, and *H*5 are verified.

### Additional robustness checks

#### Instrumental variable method

We use the IV-2SLS method for regression to avoid potential endogeneity problems in the model. We adopt the total number of words in each sample corporate’s annual report as an instrumental variable. The reason is that DT is calculated from the DT-related word frequency reflected in corporates’ annual reports. The total word count of annual reports is highly correlated with DT. And the total word count of annual reports is an exogenous model variable and has no direct causal relationship with TFP. In addition, we also chose the lag phase of DT as the instrumental variable. After testing the validity of the instrumental variables, we find that the results pass the overidentification test at the 1% level. The Cragg-Donald Wald *F*-value is greater than the critical value of the Stock-Yogo weak instrumental variable at 10%. This further indicates that the instrumental variables are reasonable. Table 4 shows the estimation results, and the conclusions are consistent with the above results.

**Table 4:** Instrumental variables method results.

	DT	TFP	DT	TFP
	First-stage	Second-stage	First-stage	Second-stage
DT		3.0862***		2.4812***
		(8.46)		(4.04)
l.DT			0.1433***	
			(8.10)	
Word	0.0008***			
	(14.55)			
FMS	0.0003*	−0.0037***	0.0004	0.0017
	(1.66)	(−2.99)	(1.29)	(1.16)
ROA	0.0002	0.0126***	0.0004**	0.0094***
	(1.39)	(15.29)	(2.27)	(11.77)
Inv	0.0003	0.0512***	0.0008	0.0534***
	(0.48)	(14.00)	(0.97)	(12.60)
RD	−0.0003	−0.0258***	−0.0004	−0.0226***
	(−0.72)	(−11.51)	(−0.77)	(−9.89)
Lev	0.0002	0.0058***	0.0002	0.0059***
	(1.55)	(8.89)	(1.61)	(7.82)
Size	0.0015	0.1770***	0.0069**	0.1590***
	(0.61)	(13.19)	(2.35)	(10.34)
Time	−0.0115	0.0455***	0.0117***	0.0541***
	(−5.06)	(3.60)	(−5.64)	(4.49)
Year	YES	YES	YES	YES
Region	YES	YES	YES	YES
*N*	4,218	4,218	3,076	3,076
*R* ^2^	0.0768	0.3590	0.0513	0.2980
*F*	24.8400***	249.8000***	11.740***	128.2000***
Cragg-Donald Wald F statistic	211.7090***		65.6080***	
Kleibergen-Paaprk Wald F statistic	199.5610***		63.9760***	

#### Sobel and bootstrap mediating effect test method

We further use the Sobel test and bootstrap method to verify the robustness of the mediating effect (Baron and Kenny, 1986). As shown in Table 5, the Sobel Z-value of 4.263 is significant at the 1% level. The confidence interval value of the bootstrap method further confirms the Sobel test results and indicates that TC plays a mediating role between DT and TFP.

**Table 5:** The results of the Sobel and bootstrap test.

Variables		Sobel Z	Boostrap (95% confidence interval)	
Dependent variable	Independent variable		*p*	BC
TFP	DT	4.263***	(0.024, 0.064)	(0.029, 0.068)

P, Percentile confidence interval BC-Bias-corrected confidence interval.

#### Replace the dependent variable and mediating variable

First, we use the LP method to measure TFP. Here, we specifically choose TFP calculated by the OLS method. Second, we replace the number of joint patent grants with the joint patent applications to measure TC. Table 6 shows the regression results; the conclusions remain consistent with the previous results.

**Table 6:** Replace variable regression results.

	lntfpols	TC	lntfpols	lntfpols	lntfpols	TFP	TC	TFP	TC	TFP
	Replace the dependent variable					Replace the mediating variable				
	(1)	(2)	(3)	(4)	(5)	(6)	(7)	(8)	(9)	(10)
DT	0.939***	0.558***	0.901***	0.244***	0.684***	0.882***	0.465***	0.868***	0.229***	0.620***
	(24.27)	(8.36)	(22.90)	(3.22)	(15.22)	(23.97)	(5.79)	(22.77)	(2.61)	(14.53)
TC			0.074***					0.053***		
			(18.64)					(14.58)		
ESG				0.005***	0.019***				0.005***	0.018***
				(5.27)	(36.53)				(4.38)	(35.95)
DT × ESG				0.037***	0.014***				0.028***	0.015***
				(4.37)	(4.26)				(3.09)	(4.73)
FMS	0.004***	0.001	0.003***	0.001	0.003***	0.003***	−0.001	0.003***	−0.001	0.0026***
	(12.49)	(1.19)	(11.19)	(0.65)	(11.87)	(10.77)	(−0.06)	(11.08)	(−0.53)	(9.96)
ROA	0.026***	0.003***	0.026***	0.002***	0.022***	0.026***	0.004***	0.026***	0.002**	0.022***
	(38.59)	(5.98)	(38.72)	(3.29)	(33.26)	(38.91)	(3.94)	(38.58)	(2.28)	(34.33)
Inv	0.0484***	−0.0016	0.0492***	−0.0002	0.0520***	0.0473***	0.0016	0.0473***	0.0028	0.0501***
	(30.34)	(−1.36)	(31.19)	(−0.13)	(36.10)	(30.45)	(0.67)	(33.07)	(1.16)	(36.87)
RD	−0.027***	0.014***	−0.029***	0.013***	−0.030***	−0.027***	0.011***	−0.029***	0.010***	−0.030***
	(−20.23)	(7.75)	(−21.23)	(7.13)	(−27.91)	(−20.88)	(4.57)	(−21.40)	(4.29)	(−25.73)
Lev	0.014***	0.002***	0.014***	0.002***	0.014***	0.014***	0.002***	0.014***	0.002***	0.014***
	(48.05)	(4.85)	(47.67)	(4.82)	(52.32)	(47.20)	(4.30)	(45.24)	(4.51)	(51.84)
Size	0.530***	0.100***	0.516***	0.066***	0.422***	0.527***	0.101***	0.518***	0.068***	0.425***
	(124.86)	(17.31)	(127.51)	(10.59)	(95.38)	(126.49)	(14.60)	(123.26)	(9.75)	(98.35)
Time	0.005***	0.004***	0.005***	0.002*	0.004***	0.005***	0.002	0.004***	0.001	0.004***
	(7.83)	(3.14)	(6.88)	(1.86)	(5.76)	(7.28)	(1.56)	(6.96)	(0.48)	(6.04)
Year	YES	YES	YES	YES	YES	YES	YES	YES	YES	YES
Region	YES	YES	YES	YES	YES	YES	YES	YES	YES	YES
_cons	−0.739***	−2.061***	−0.451***	−1.373***	1.295***	−0.641***	−1.980***	−0.456***	−1.334***	1.250***
	(−8.75)	(−17.33)	(−5.44)	(−11.02)	(15.04)	(−7.73)	(−14.22)	(−5.42)	(−9.75)	(14.73)
N	4,222	4,222	4,222	4,222	4,222	4,222	4,222	4,222	4,222	4,222
Wald chi2	49772.860***	825.840***	57748.050***	842.860***	75670.770***	53439.560***	567.270***	54192.280***	589.090***	76253.230***

## Discussion and conclusion

This study aimed to investigate how DT influences TFP through the mediating role of TC and the moderating role of ESG. Our research finds that DT can boost corporate TFP, which is consistent with Ballestar et al. (2021) empirical work that the existence of long-term productivity-augmenting effects as a result of the implementation of DT in Spanish manufacturing firms. Our finding also provides further evidence for the Zeng and Lei (2021) argument that DT can improve TFP through technological advances that lead to efficient production models and data-driven intelligent and efficient production. However, the results of their research provide empirical evidence of the impact of DT corporate TFP but fail to link this indirect effect. This paper further explores the mechanism of DT and TFP through TC. We find that DT can boost TC between corporate and other organizations.

Moreover, TC acts as a mediator in DT to improve TFP. On the one hand, the findings support Wang et al. (2018) argument that IT could facilitate corporate collaborative innovation. However, these studies emphasize the impact of specific digital technologies on inter-firm technological cooperation. DT mainly relies on big data, digital platforms, and other technologies to realize the change from industrial management mode to digital management mode. We have further integrated the impact of DT on TC. On the other hand, we find that DT can effectively break down spatial constraints and strengthen the connection between corporates and external organizations. Firms form an innovative synergy through TC, which helps them increase innovation capacity, reduce production costs, and improve product quality (Singh et al., 2022), further leading to increased corporate TFP. The findings support Gaglio et al. (2022) argument that innovation conditional based on the DT has a positive effect on corporate productivity in South Africa. It is also in line with Mohnen et al. (2018) empirically found that ICT is complementary to corporate innovation as joint investment leads to higher TFP in the Netherlands. Contrary to these studies that have demonstrated the impact of innovation with DT on corporate productivity, we further highlight the innovative forms of TC play an important mediating role between DT and TFP.

Our findings suggest that ESG performance is positively moderating in DT and TC. This is in line with Broadstock et al. (2021b) who argued that ESG increases the ability of corporates to pursue innovative activities, ultimately positively affecting their value creation and financial performance. However, these studies have mainly emphasized the direct impact of ESG on corporates” innovation or performance or the interaction between ESG and innovation (Jung et al., 2022). Our study extends their findings by providing further evidence on the moderating role of ESG on innovation activities in TC. In addition, Alkaraan et al. (2021) found a moderating role of ESG between corporate transformation toward Industry 4.0 and financial performance in the United Kingdom context. We further support their study on this basis with a DT perspective, suggesting that ESG also plays a moderating role in DT and corporate and TFP, providing firm-level evidence for ESG moderating role research in China.

### Theoretical implications

The theoretical contributions of our study are threefold. First. Based on IPT theory (Galbraith, 1973). Previous studies have mainly documented ICT in corporate productivity (Brynjolfsson and Hitt, 2002; Pieri et al., 2018; Pan et al., 2022). However, manufacturing corporates tend to integrate and configure these front-end technologies to establish DT, thus improving corporate management efficiency from multiple aspects to remove barriers to support their operations and environmental management (Ivanov et al., 2019). On the basics, our study found that DT can boost corporate TFP. It helps corporates collect and process information efficiently, expand their scale efficiency, and achieve data-driven intelligent and efficient production. Our study helps to enhance the current understanding of the antecedents of TFP and enriches the existing literature on DT in microeconomic subjects. This study’s contribution is vital for future enterprise productivity, as DT is an essential driver of TFP. Meanwhile, the empirical findings are consistent with the IPT perspective, suggesting that IPT is a valuable way to characterize DT’s effects and contextual conditions for TFP.

Second, Previous studies have mainly documented the adoption of digital technologies in a corporate collaborative innovation (Wang et al., 2018). They have not examined the mediating role of TC as the mechanism explaining the relationship between DT and TFP. Our study shows that DT reduces barriers to TC and reshapes the way value creation between corporates and other organizations. Corporate TC is an effective measure to improve TFP. Our study better explains the impact of DT on corporate TFP and extends the existing literature on TC.

Third, we identify the moderating role of ESG performance on the impact of DT on TC and TFP. However, to the best of our knowledge, there is very limited empirical research on the moderating effect of ESG in corporate DT. From the perspective of social responsibility theory, this study is one of the early studies to establish a union between IPT and social responsibility theory. We found that ESG enables corporates to maintain good relationships with multiple stakeholders and reduce information asymmetries. It helps corporates create a good reputation (Chouaibi et al., 2021) and gain the technology partner’s trust (Fatemi et al., 2015). At the same time, ESG can alleviate principal-agent problems and reduce environmental violation costs and financing constraints. It further supports the positive effects of DT on TC and TFP. This paper provides a more comprehensive understanding of corporate ESG in generating economic outcomes in complex social systems.

### Practical implications

Corporates should be sensitive to the development opportunities in DT and accelerate the introduction of digital technologies. It is recommended that corporates use digital technology to transform traditional production methods, promote the comprehensive docking of internal manufacturing resources and Internet platforms, and strengthen the efficiency-enhancing effects of DT. Government should create a favorable external environment, such as introducing targeted financial or tax policies for corporate DT. Improve laws and regulations to address the new characteristics of infringement in the digital economy era. Increase the protection of intellectual property rights of digital technology and data assets. Optimize the market management system and administrative approval process related to corporate transformation and clear various external obstacles enterprises face in DT.

Corporates should pay attention to the role of TC in production. Expand the breadth of R&D partners and take the initiative to cooperate with partners of different organization types such as customers, suppliers, universities, or research institutes. In this way, corporates can build multi-party participation, long-term stable technology partnership and gradually form a diversified R&D cooperation. For example, corporates can expand their knowledge base by co-constructing R&D platforms, holding technical exchanges, and building laboratories with universities and research institutes. Thus, the advantages between different innovation organizations are complementary, and the resources are linked to carrying out R&D activities jointly. In addition, the government should take measures to lower the threshold of cooperation between enterprises and other organizations. Build an innovative ecological network by creating an online information matching platform or creating a joint committee for TC. Encourage sharing and co-creation between corporates and other organizations., and promote the in-depth exchange of talents, technologies, and information among different organizations.

Corporates should enhance ESG awareness and treat it as a value investment rather than a cost investment. In particular, corporates should improve the top-level design of ESG, cultivate environmental awareness, actively assume social responsibility, strengthen internal corporate governance, and implement ESG concepts into all aspects of corporate development. Meanwhile, corporates should take the initiative to strengthen ESG information disclosure and establish a good reputation image. The government should guide corporates to strengthen ESG information disclosure, increase the cost of poor ESG performance or false performance, and provide specialized ESG advisory services to corporates. At the same time, investors should incorporate ESG into the decision-making framework. To reduce investment risks, they should also pay attention to non-financial information such as environmental responsibility, social responsibility, and corporate governance. And investors’ attention to ESG performance will also guide the benign development of corporates.

### Limitations and future research

This study has some limitations that can guide future research. First, the sample is drawn from the manufacturing industry and does not involve other industries. Therefore, future research should expand to include samples from other industries. Second, since the effect of DT on corporate TFP may be all-around, and we only focus on the role of TC and ESG, future research can explore other possible effect mechanisms such as corporate technology frontier distance or the heterogeneity effect between DT and TFP due to different geographical locations.

## Data availability statement

The raw data supporting the conclusions of this article will be made available by the authors, without undue reservation.

## Author contributions

NL performed conceptualization, methodology, visualization, and writing-original draft. XW did conceptualization, methodology and validation. ZW and XL were involved in conceptualization, methodology, validation, and writing-review and editing. All authors contributed to the article and approved the submitted version.

## Funding

This paper received funding from the National Natural Science Foundation of China “Research on the Impact of University-Industry Cooperation on Research Performance of Chinese Universities” (project number: 71874042). The Fundamental Research Funds for the Central Universities (project number: HIT.HSS.202102).

## Conflict of interest

The authors declare that the research was conducted in the absence of any commercial or financial relationships that could be construed as a potential conflict of interest.

## Publisher’s note

All claims expressed in this article are solely those of the authors and do not necessarily represent those of their affiliated organizations, or those of the publisher, the editors and the reviewers. Any product that may be evaluated in this article, or claim that may be made by its manufacturer, is not guaranteed or endorsed by the publisher.
